# A novel method to remove impulse noise from atomic force microscopy images based on Bayesian compressed sensing

**DOI:** 10.3762/bjnano.10.225

**Published:** 2019-11-28

**Authors:** Yingxu Zhang, Yingzi Li, Zihang Song, Zhenyu Wang, Jianqiang Qian, Junen Yao

**Affiliations:** 1 School of Instrumentation and Optoelectronic Engineering, Beihang University, Beijing 100191, China; 2 Key Laboratory of Micro-nano Measurement-Manipulation and Physics (Ministry of Education), Beihang University, Beijing 100191, China; 3 School of Physics, Beihang University, Beijing 100191, China

**Keywords:** atomic force microscopy (AFM), Bayesian compressed sensing, denoising, image processing, impulse noise

## Abstract

A novel method based on Bayesian compressed sensing is proposed to remove impulse noise from atomic force microscopy (AFM) images. The image denoising problem is transformed into a compressed sensing imaging problem of the AFM. First, two different ways, including interval approach and self-comparison approach, are applied to identify the noisy pixels. An undersampled AFM image is generated by removing the noisy pixels from the image. Second, a series of measurement matrices, all of which are identity matrices with some rows removed, are constructed by recording the position of the noise-free pixels. Third, the Bayesian compressed sensing reconstruction algorithm is applied to recover the image. Different from traditional compressed sensing reconstruction methods in AFM, each row of the AFM image is reconstructed separately in the proposed method, which will not reduce the quality of the reconstructed image. The denoising experiments are conducted to demonstrate that the proposed method can remove the impulse noise from AFM images while preserving the details of the image. Compared with other methods, the proposed method is robust and its performance is not influenced by the noise density in a certain range.

## Introduction

Atomic force microscopy (AFM) is a powerful tool in the fields of nanoscience and nanotechnology, because it can be used to acquire high-resolution images of all kinds of samples in various environments. Nowadays, AFM is also used to acquire information about the physical properties of the samples [1]. The quality of the images is of fundamental importance for acquiring information about structure and properties of the sample surface at the nanoscale. The more details are shown by the image, the more information about the sample will be obtained. However, noise that comes from the environment and the instrument itself will reduce the quality of the AFM images, and details of the image may be hidden in the noise [2].

There is no method that can filter out all types of noise at the same time. Therefore, different filtering methods, according to the type of the noise, need to be applied to acquire high-quality images with removed noise [3–4]. Chen [5] has proposed “unsupervised destripe” to remove the non-uniform stripe noises from AFM images. Orthogonal wavelets are applied to filter the Gaussian noise from AFM images [6]. For the impulse noise in AFM images, the median filter is generally applied [7–8], where every pixel is replaced by the median value of pixels of the neighborhood. In order to reduce the image blurring and the loss of details, the adaptive median filter [9] is proposed where only the noisy pixels are replaced. The removal of impulse noise is decomposed into two steps: the identification of noisy pixels and the recovery of the true image. To further improve the denoising performance, machine learning [10] and neural networks [11–12] are introduced to help remove the impulse noise. First, machine learning or neural networks are used to improve the accuracy of the recognition of noisy pixels. Then, the noise pixels are replaced by the median value. The key to improve the filtering effect of these methods is to more accurately identify the noisy pixels. The performance of the filtering will decrease as the impulse noise density increases [13]. It is noteworthy that an image with the noisy pixels removed can be seen as an undersampled image that is generated through compressed sensing (CS) sampling. Therefore, compressed sensing can be introduced for the removal of impulse noise. If the sampling rate is higher than the sparsity of the original signal, the original signal can be recovered through compressed sensing [14]. This means that the noise removal does not degrade the image quality.

**Figure 1:**
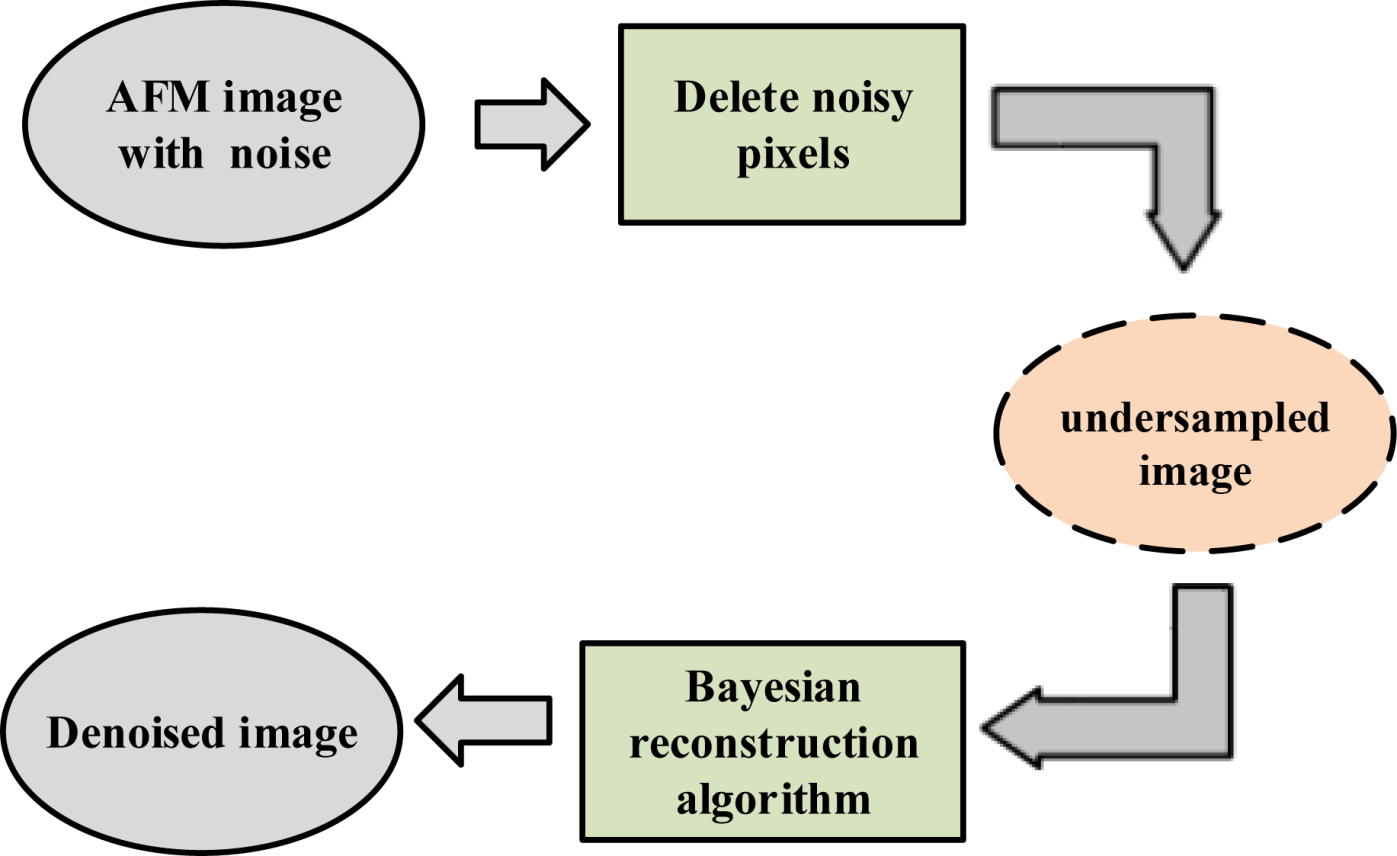
The schematic of removing impulse noise from AFM images based on Bayesian compressed sensing.

CS is an established way to use few compressed data to represent high-dimensional data. It has been used to recover the signal from data sampled far below the classic Nyquist sampling rate under certain conditions [15–16]. Generally, the purpose of introducing CS into AFM is to increase the imaging rate [17–19]. The essentials for applying CS in AFM are the sparse representation of the image, the generation of a measurement matrix and the design of a reconstruction algorithm. For the AFM image, it will show sparsity in an orthogonal transform domain, although it is not sparse in the spatial domain. The identification process of noisy pixels can be seen as compressed sampling. However, it is impossible to ensure that the process meets the demand of theoretical reconstruction guarantees [20–21]. Bayesian compressed sensing (BCS) [22] provides a better reconstruction performance than other methods when the theoretical reconstruction guarantees are partially destroyed [23]. Different from other reconstruction methods, BCS does not need the acquisition of the sparsity of the original signal and can achieve a good reconstruction even when the original signal is not sparse [14]. In addition, AFM tracks the sample line-by-line to obtain the image, which means that the acquisition process of two adjacent lines of the image can be regarded as irrelevant. Therefore, a novel method to remove impulse noise for AFM image using BCS is obtained according to the AFM imaging characteristics, shown as Figure 1.

Here, a novel method based on BCS is proposed to remove the impulse noise from AFM images. First, the proposed method transforms the removal of impulse noise from AFM images into a problem of CS imaging in AFM, and the details of the method are presented. Then, denoising experiments are shown to demonstrate that the proposed method can remove the impulse noise while retaining the details of the image. In addition, the proposed method is robust and its performance will not be affected by the impulse noise density within a certain range. Finally, detailed discussions about the proposed method are given.

## Details of Denoising through Bayesian Compressed Sensing

### Introduction of compressed sensing in AFM

Usually, the AFM tracks a sample following a raster scan mode and every pixel of the image is sampled independently. Therefore, a *n* × *n* AFM image can be seen as a 2D matrix and all rows of the matrix are stacked together to generate a long vector **x**^T^ ∈ R*^N^*^×1^ (*N* = *n* × *n*), where *N* is the total number of pixels of the AFM image. If the AFM conducts *M* samplings, the process of the AFM imaging can be expressed as

**Figure 2:**
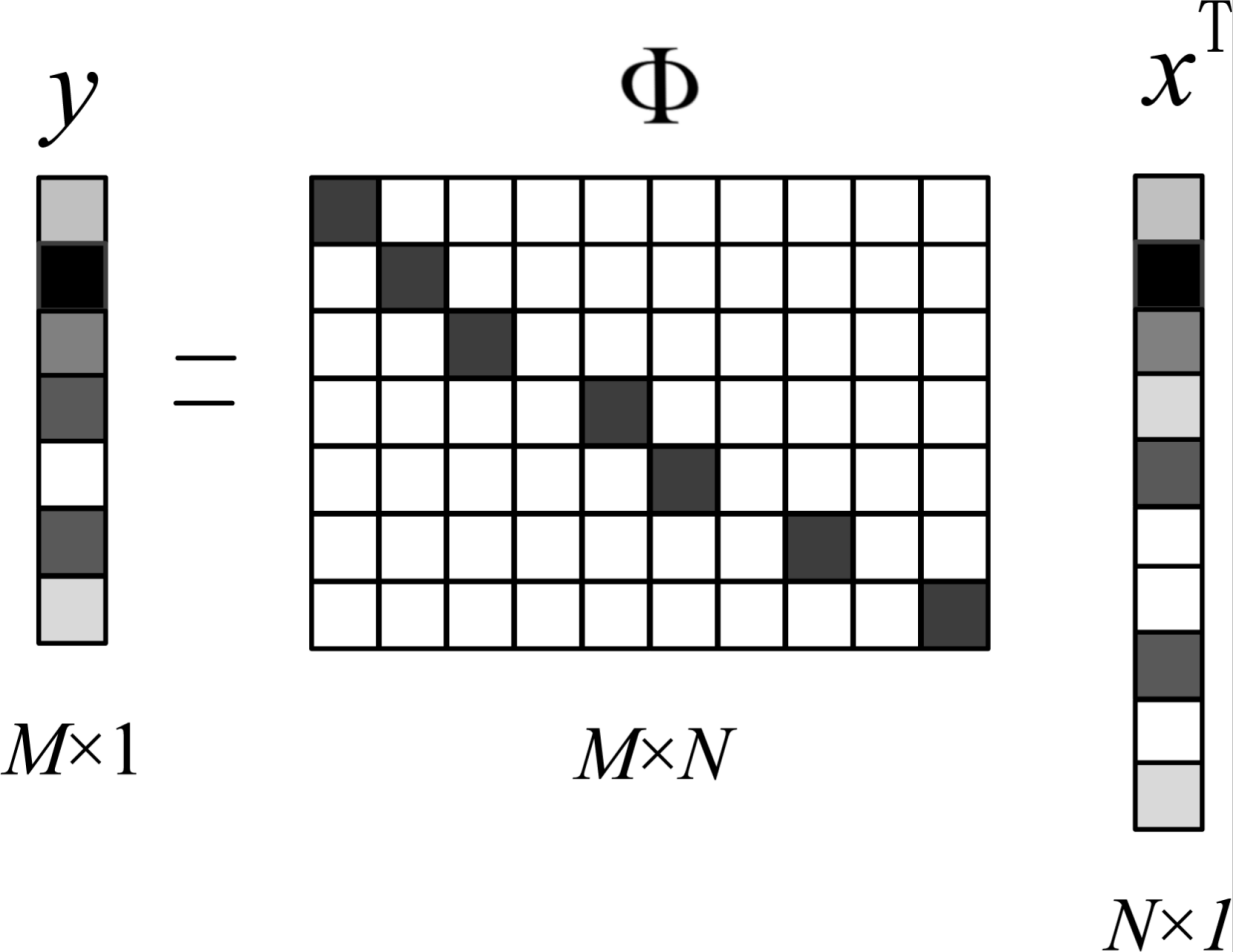
Mathematical model of compressed sensing AFM imaging.

[1]
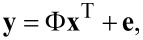


where **y**∈ R*^M^*^×1^ is the measurement result, Φ ∈ R*^M^*^×^*^N^* is the measurement matrix, **x**^T^ ∈ R*^N^*^×1^ represents the true sample topography, and **e** is the measurement noise. If *M* is smaller than *N*, then the AFM imaging is CS imaging. Because the AFM only samples one pixel of an image at a time and moves around to obtain an image, the compressed sampling of the AFM can be seen as randomly collecting partial elements of **x**. The undersampling process can be modeled by an identity matrix with some rows removed, shown in Figure 2. In order to recover the true AFM image from the undersampled data, the sample topography **x** must be sparse, i.e. some elements **x** must be zero, and the measurement matrix Φ must meet certain constraint conditions [24].

However, an AFM image cannot be sparse in real space. A sparse dictionary Ψ ∈ R*^N^*^×^*^N^* is introduced to improve its sparsity [25]. Thus, **x**^T^ can be expressed as **x**^T^ = Ψα (α ∈ R*^N^*^×1^) and α is sparse, i.e., some elements of α are zero. Equation 1 can be rewritten as

[2]
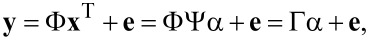


where Γ = Φ × Ψ ∈ R*^N^*^×^*^N^*. Since the number of measurements *M* is smaller than the number of unknowns *N*, Equation 2 cannot be solved directly. Because there are some zero elements in α, the solution of Equation 2 can be expressed as an *L*_0_-norm optimization problem

[3]
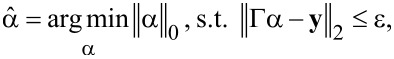


where ε is the bound of the noise. The solution of Equation 3 is a non-deterministic polynomial problem (NP problem) [26]. When both the number of total elements of α and its non-zero elements are large, a solution of Equation 3 cannot be achieved in practice. Generally, the non-convex *L*_0_-norm can be approximated by a convex *L*_1_-norm [27],

[4]
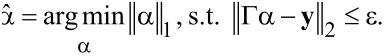


Now, solving Equation 2 is transformed to an *L*_1_-norm minimization.

### Identifying and deleting noisy pixels

The problem of impulse noise removal has been transformed into a problem of compressed sensing. Therefore, the first thing is to obtain the measurement matrix and the undersampled image, which can be achieved by identifying and removing the noisy pixels. There are two ways, including interval approach and self-comparison approach, to identify noisy pixels. For the interval approach, noisy pixels can be identified according to

[5]
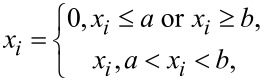


where *a* is the lower limit of the pixel value and *b* is the upper limit. A pixel *x_i_* out of the range (a,b) will be regarded as noisy pixel and will be defined as 0. After all elements have been checked according to Equation 5, the elements defined as 0 will be removed directly to generate an undersampled image. When the distribution of noise intensity is relatively narrow, the noisy pixels can be identified easily by setting a lower limit *a* and an upper limit *b*. Although some regular pixels may be mistaken for noisy pixels, this will not reduce the quality of the denoised image [14]. When the distribution of the noise intensity is dispersive, appropriately increasing the lower limit while decreasing the upper limit allows for more noisy pixels to be identified. However, this may also cause more regular pixels to be removed by mistake, and even a large number of pixels in a certain area might be deleted. If there are too many regular pixels removed by mistake, the quality of the denoised image will drop because of the loss of image information. Therefore, the range of the interval should be set carefully.

The self-comparison approach is another way to identify noisy pixels. Noisy pixels are identified by comparing the target pixel with its neighborhood. At first, an *r* × *r* (*r < n*) area of the image with the target pixel in the center is chosen, where *x*_max_ and the *x*_min_ are the maximum pixel value and minimum pixel value in the area, respectively. Then, the target pixel value *x_i_* (*i* = 1,…, *N*) will be compared with *x*_max_ and *x*_min_ according to

[6]
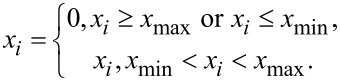


If the pixel *x_i_* is exactly the maximum value or the minimum value of the area, it will be regarded as a noisy pixel and defined as 0. All pixels of the image will be checked according to Equation 6. After all the pixels were checked, the elements defined as 0 will be removed directly. The self-comparison approach avoids that a large number of pixels in the same area is removed by mistake. Although some regular pixels might be deleted by mistake especially for pixels with large or small values, the quality of the denoised image will not be reduced because of the robustness of the proposed method. However, if there are a lot of flat areas in the image, the approach may delete regular pixels incorrectly, and its performance will be worse than that of the interval approach.

When CS is applied to AFM imaging, every row of the AFM image is stacked together to build a 1D vector **x**^T^. Although a 2D denoised image is obtained, the reconstructed result directly obtained from AFM CS imaging is a 1D vector of dimension *N*. Therefore, the neighborhood of the target pixel has been changed to a 1D interval with a size of *r*. However, converting an image to a 1D vector will lose 2D structural information, which means it may not be the best choice to treat the image as a vector. Because the AFM tracks the sample line-by-line, which means the acquisition process for each row is independent, treating the image as a vector will not lose image information. In addition, the end of one row is independent of the beginning of its adjacent row. Therefore, the self-comparison can be conducted line-by-line independently. Thus, an undersampled image is obtained by removing the noisy pixels.

The removal of noisy pixels can be regarded as compressed sampling. There are only two possibilities for every pixel. It is either a noisy pixel or a noise-free pixel. The noisy pixels are removed and the noise-free pixels are preserved. Therefore, the elements of the measurement matrix Φ only consist of 1 and 0, which is the same as the AFM CS imaging. If there is no impulse noise in the image, the measurement matrix Φ will be an identity matrix. Otherwise, the corresponding rows of the identity matrix need to be removed, as shown in Figure 2. Because the identification of the noisy pixels is performed line-by-line, the measurement matrix Φ should be constructed according to the corresponding row. Thus, the process of impulse noise removal can be described by Equation 2. *n* measurement matrices and measurement results will be generated for an AFM image with a size of *n* × *n*.

### Fast reconstruction of the image

Usually, there will be only one measurement matrix Φ and one measurement result **y** obtained for the AFM CS imaging because all the rows of the image are stacked together to generate a vector [19,28]. In CS AFM imaging, recovering the true sample topography from the undersampled information has high computational cost. For a *n* × *n* AFM image, the compressed sampling matrix is *M* × *N* (*n* ≪ *M < N*), which requires a lot of memory space and computing resources [29]. For a typical AFM image with a resolution of 256 × 256 pixels, only the matrix will occupy 32GB RAM and the reconstruction time exceeds 1 h on a server. If the resolution of the AFM image continues to increase, the true sample topography can not be recovered easily. Therefore, the traditional reconstruction methods applied in AFM imaging are not suitable to recover the AFM image obtained from removing the noisy pixels. In order to remove the impulse noise in the AFM image quickly, a faster reconstruction of the image needs to be achieved.

If each row of the AFM image is regarded as a sub-vector, these sub-vectors are independent of each other, because they are simply stacked together. In addition, the AFM tracks the sample surface line-by-line to measure the topography, which means that the acquisition process of two adjacent lines of the image is independent. Because the tracking of one line is hardly affected by the adjacent tracking line, every tracking line of the AFM image can be compressed sampled and recovered independently. Therefore, the reconstruction of the AFM image can be decomposed into the reconstruction of a series of vectors. Recovering the image from undersampled AFM data line-by-line will not reduce the quality of the image compared with the traditional method. After the removal of noisy pixels, *n* measurement matrices and results will be obtained, which means each row will be processed independently. In order to obtain a denoised image, the reconstruction will be performed *n* times.

CS provides a lot of methods to recover the original signal, such as basis pursuit [30], iterative hard thresholding [31], orthogonal matching pursuit [32] and Bayesian compressed sensing [33]. The measurement matrix generated by recording the position of the noise-free pixels may not fully meet theoretical reconstruction guarantees. In our previous work [34], which aims to develop a fast AFM image reconstruction from undersampled AFM data, BCS has been proven to be a better method to reconstruct AFM images from undersampled AFM data than other methods. There is also no guarantee that the measurement matrix obtained from the removal of noisy pixels will fully satisfy theoretical reconstruction guarantees. The developed reconstruction algorithm can be used to recover the image. The details of the BCS reconstruction algorithm in AFM are given in our previous work [34].

## Results and Discussion

Denoising experiments are conducted to evaluate the performance of the proposed method. Impulse noise is added to AFM images to generate noisy images. The AFM images are converted to 8-bit grayscale images, and the resolution of the AFM images used in the paper is 256 × 256 pixels. The proposed method, median filter (window size: 7 × 7 pixels) and adaptive median filter (maximum window size: 7 × 7 pixels, minimum window: 3 × 3 pixels) are used separately to remove the impulse noise. The peak signal-to-noise ratio (PSNR) [35] and the structural similarity (SSIM) index [36] are used to evaluate quantitatively the performance of the filtering. The details of PSNR and SSIM can be found in [37]. The AFM image before adding impulse noise is considered as the standard image, and the values of PSNR and SSIM are obtained through comparing the denoised image with the standard image. If the value of PSNR is greater than 35 dB, the quality of the denoised image is very good and the distortion is negligible. When the PSNR is less than 30 dB, the performance of the denoising is poor and the distortion of the image is not negligible. When the SSIM is greater than 0.9, the denoising is acceptable regarding the visual quality.

**Figure 3:**
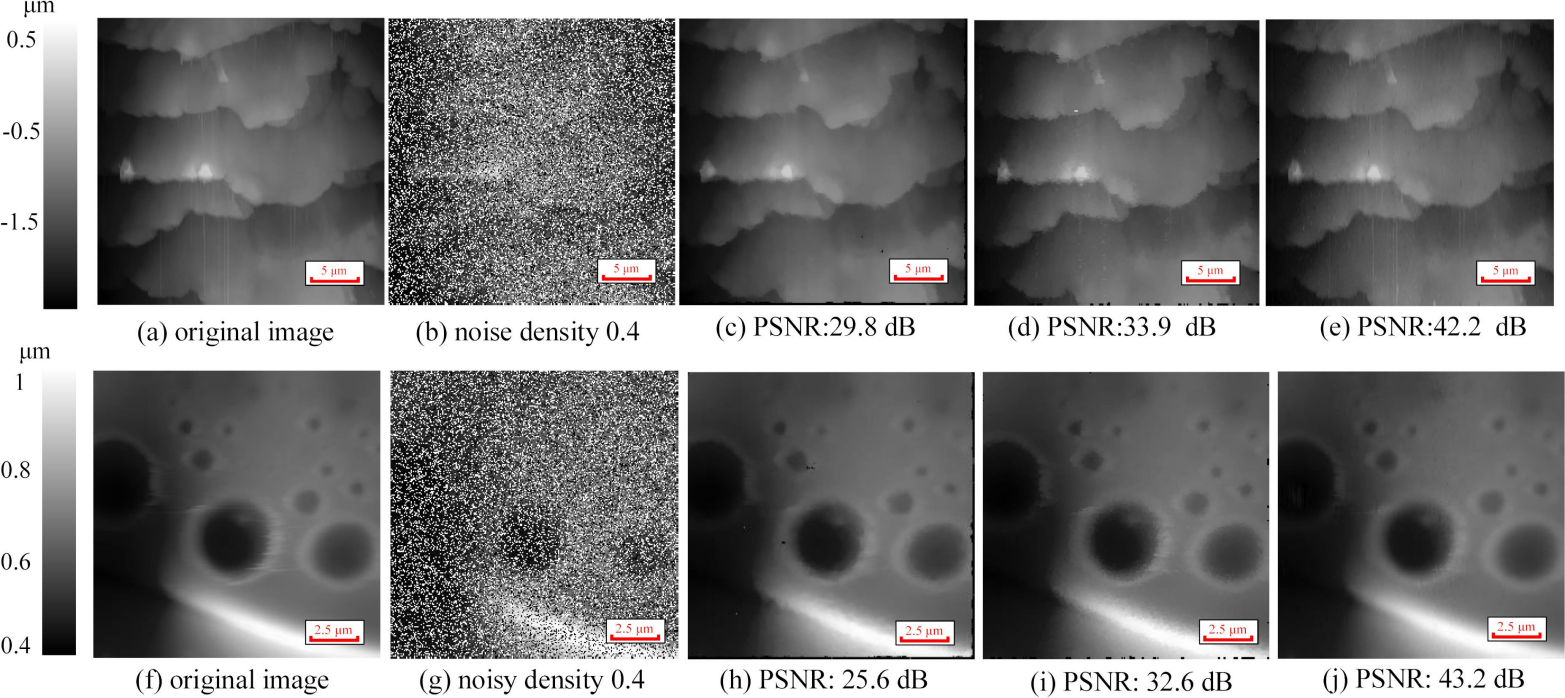
Comparison of denoising between the interval-BCS denoising, the median filter and the adaptive median filter. (a, f) Original images. (b, g) Images with added impulse noise with a density of 0.4. (c) Denoised image obtained by the median filter; the PSNR is 29.8 dB, and the SSIM is 0.92. (d) Denoised image obtained by the adaptive median filter; the PSNR is 33.9 dB, and the SSIM is 0.95. (e) Denoised image obtained by the interval-BCS denoising, where the upper limit is 254 and the lower limit is 5. Here, the PSNR is 42.2 dB, and the SSIM is 0.96. The runtime is 6.7 s. (h) Denoised image obtained by the median filter; the PSNR is 25.4 dB, and the SSIM is 0.93. (i) Denoised image obtained by the adaptive median filter; the PSNR is 32.6 dB, and the SSIM is 0.96. (j)Denoised image obtained by the interval-BCS denoising, where the upper limit is 250 and the lower limit is 5. Here, the PSNR is 43.2 dB, and the SSIM is 0.97. The runtime is 8.3 s.

The denoising performance of the proposed method using two different noise identifying approaches are tested separately. First, BCS denoising using the interval approach (interval-BCS denoising) is used to remove the impulse noise, and the denoised images are compared with those obtained by applying the median filter and the adaptive median filter. The experiments are conducted in Matlab on a personal computer (Intel Core i5, 8 GB RAM, Windows 10 x64). Impulse noise with a density of 0.4 is added to the images. The results are shown in Figure 3. It can be seen from Figure 3c–e and Figure 3h–j that all the pixels with impulse noise have been removed from the AFM image while details of the image are preserved in the denoised image. The denoised images (Figure 3e,j) obtained by the proposed method are almost the same as the original images (Figure 3a,f). In addition, the values of PSNR of the denoised image obtained by interval-BCS denoising are greater than 35 dB, which means the distortion caused by denoising is negligible. The PSNR value obtained by the median filter is less than 30 dB, which means that non-negligible distortion has occurred. The PSNR value obtained by the adaptive median filter is larger than 30 dB but less than 35 dB. The SSIM values obtained by the interval-BCS denoising, the median filter and the adaptive median filter are more than 0.9, which means all methods remove the impulse noise successfully from the perspective of visual quality. The interval-BCS denoising removes the impulse noise from the AFM image successfully while preserving the details, and the image distortion caused by the proposed method is negligible.

**Figure 4:**
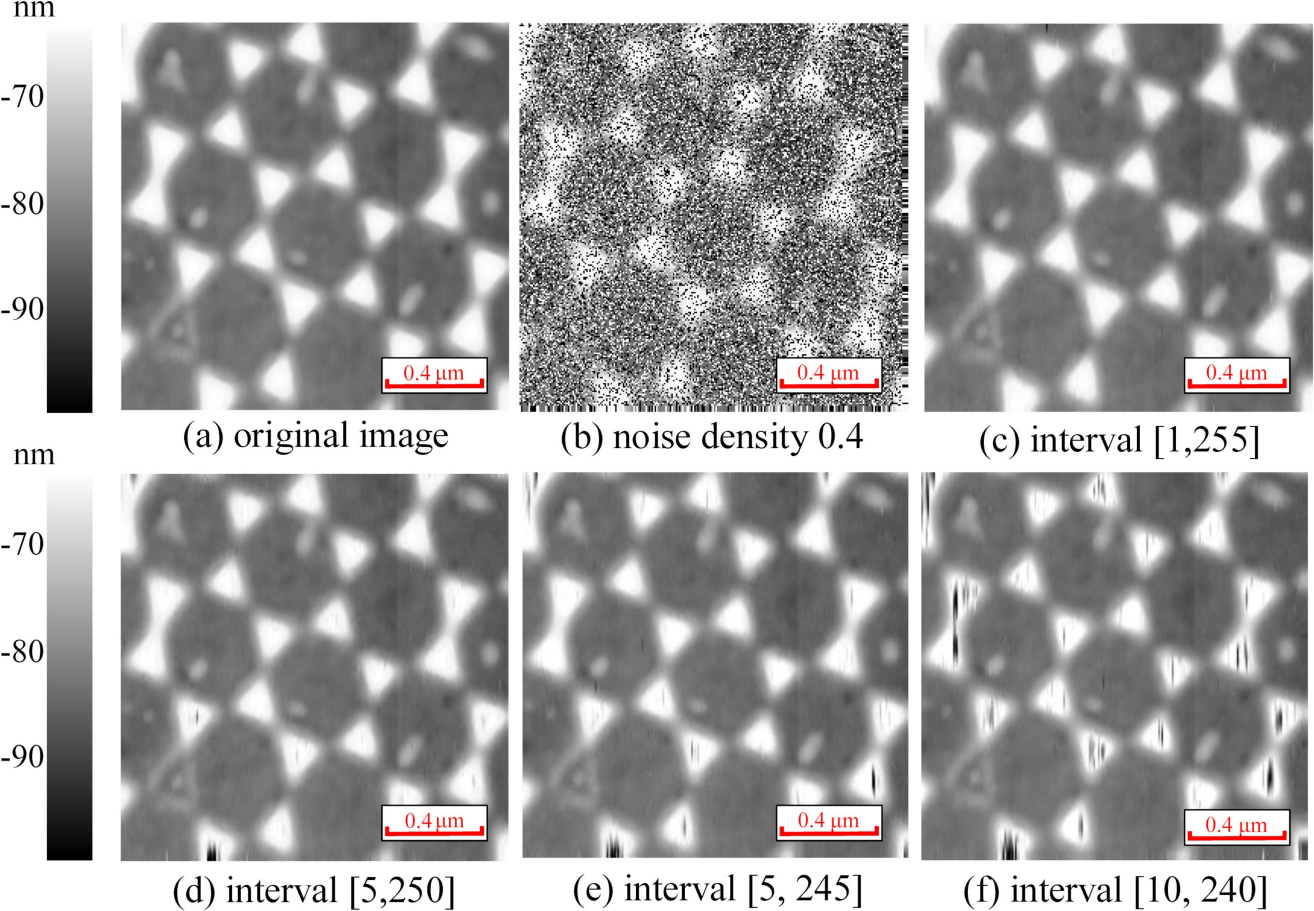
Comparison of interval-BCS denoising with different upper and lower limits. (a) Original image. (b) Image with added impulse noise with a density of 0.4. (c) Upper limit of 255 and lower limit of 1; PSNR = 40.8 dB, SSIM = 0.97, the runtime is 9.4 s. (d) Upper limit of 250 and lower limit of 5; PSNR = 33.3 dB, SSIM = 0.96, the runtime is 9.4 s. (e) Upper limit of 245 and lower limit of 5; PSNR = 29.3 dB, SSIM = 0.94, the runtime is 9.7 s. (f) Upper limit of 240 and lower limit of 10; PSNR = 23.1 dB, SSIM = 0.91, the runtime is 9.7 s.

For the interval-BCS denoising, the two parameters upper limit and lower limit must be set. Different upper limits and lower limits are used to remove the impulse noise in the Figure 3b and Figure 3g. Figure 3e was obtained with an upper limit of 254 and a lower limit of 5, and Figure 3j was obtained with an upper limit of 250 and a lower limit of 5. Inappropriate upper and lower limits will cause regular pixels in a certain area to be removed, which may cause a drastic drop in the image quality. In Figure 4, different upper and lower limits are used. Although the impulse noise in Figure 4b was removed successfully with different upper and lower limits, the loss of image details can be seen clearly in Figure 4d–f. If the noisy pixels are close to the values of noise-free pixels, it is difficult to chose suitable upper and lower limits to distinguish noise-free pixels and noisy pixels.

**Figure 5:**
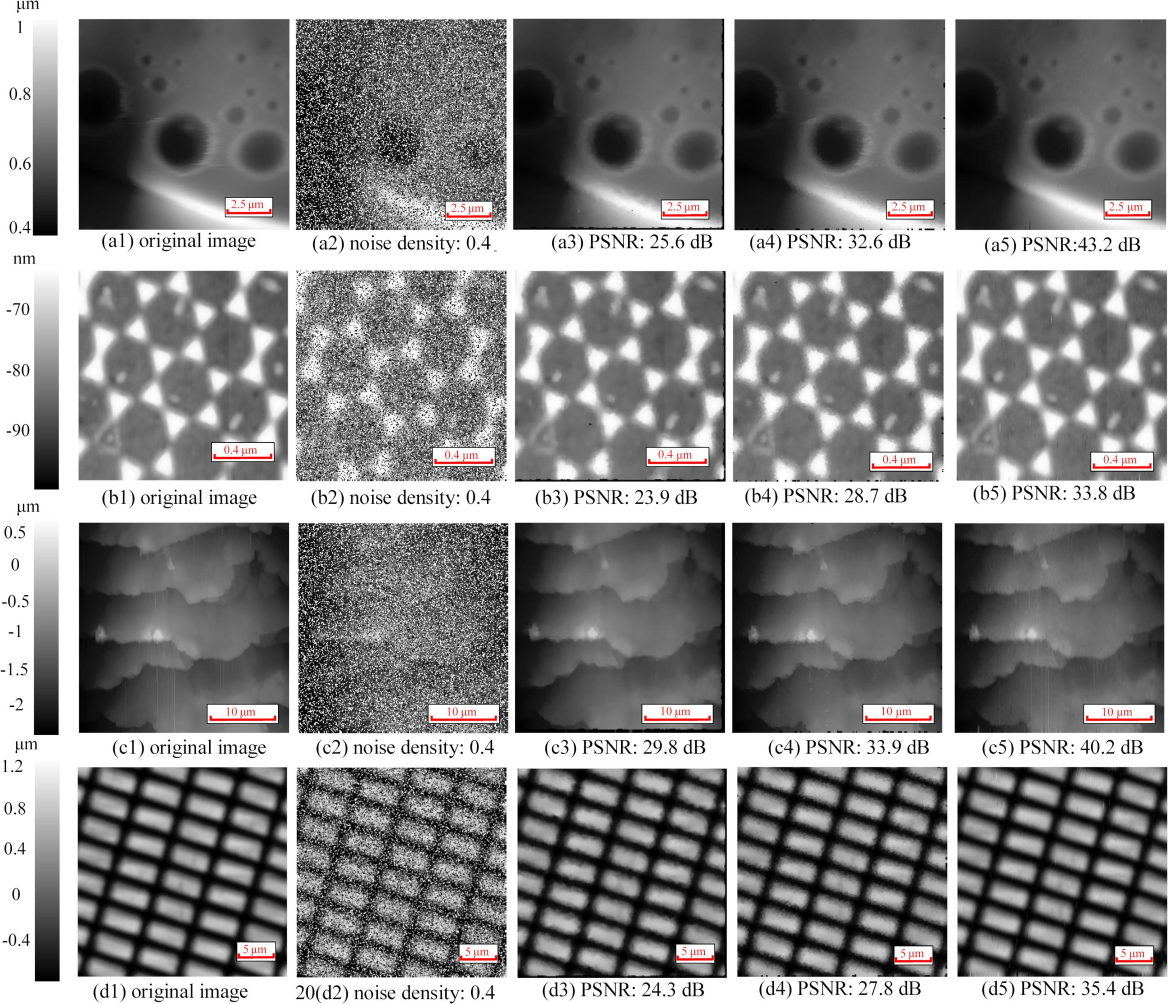
Denoised images. (a1–d1) Original images. (a2–d2) Images with added impulse noise with a density of 0.4. (a3–d3) Denoised images obtained by the median filter. (a4–d4) Denoised images obtained by the adaptive median filter. (a5–d5) Denoised images obtained by the self-comparison-BCS denoising.

The images obtained by BCS denoising using the self-comparison approach (self-comparison-BCS denoising) are shown in Figure 5. According to Equation 6, the comparison neighborhood is set as [−4,4], where the position of the target pixel is the origin. It can be seen that the impulse noise in Figure 5a2–d2 has been removed while details of the images are presented clearly. The images (Figure 5a5–d5) obtained by the self-comparison-BCS denoising are almost the same as the original images (Figure 5a1–d1). The PSNR values of the denoised images obtained by the self-comparison-BCS denoising are also greater than 35 dB (except in Figure 5b5), which means the image distortion caused by self-comparison-BCS denoising is negligible. However, there are non-negligible distortions occurring in the images processed by the median filter with PSNR values below 30 dB. And the PSNR values of the images acquired by the adaptive median are below 35 dB (some are less than 30 dB), which means its performance is also worse than that of the self-comparison-BCS denoising. In addition, the SSIM values obtained by the interval-BCS denoising, the median filter and the adaptive median filter are similar. Therefore, the self-comparison-BCS denoising removes successfully the impulse noise from AFM images while preserving details.

The comparison of self-comparison-BCS denoising and the interval-BCS denoising is shown in Table 1. It can be seen that the interval-BCS denoising and the self-comparison-BCS denoising yiled almost the same results regarding the removal of impulse noise from AFM images. When the image contains periodic structures and flat areas, the performance of the interval-BCS denoising is better than that of the self-comparison-BCS denoising, as shown in Table 1. For the noisy image in Figure 5c2, the performance of the interval-BCS denoising is better than that of the self-comparison-BCS denoising. The self-comparison-BCS denoising may remove noise-free pixels incorrectly in the flat area, because pixels in the flat area have nearly the same values.

**Table 1:** Denoising results of the images in Figure 5. “Self” refers to the self-comparison-BCS denoising, “interval” refers to the interval-BCS denoising, “median” refers to the median filter, and “adaptive” refers to the adaptive median filter.

Figure	PSNR (dB)	PSNR (dB)	PSNR (dB)	PSNR (dB)	SSIM	SSIM	SSIM	SSIM
	(self)	(interval)	(median)	(adaptive)	(self)	(interval)	(median)	(adaptive)

a1	43.2	43.2	25.6	32.6	0.97	0.97	0.93	0.96
b1	33.8	40.8	23.9	28.7	0.96	0.97	0.89	0.92
c1	40.2	41.0	29.8	33.9	0.95	0.95	0.92	0.94
d1	35.4	36.4	24.3	27.8	0.95	0.96	0.89	0.91

**Figure 6:**
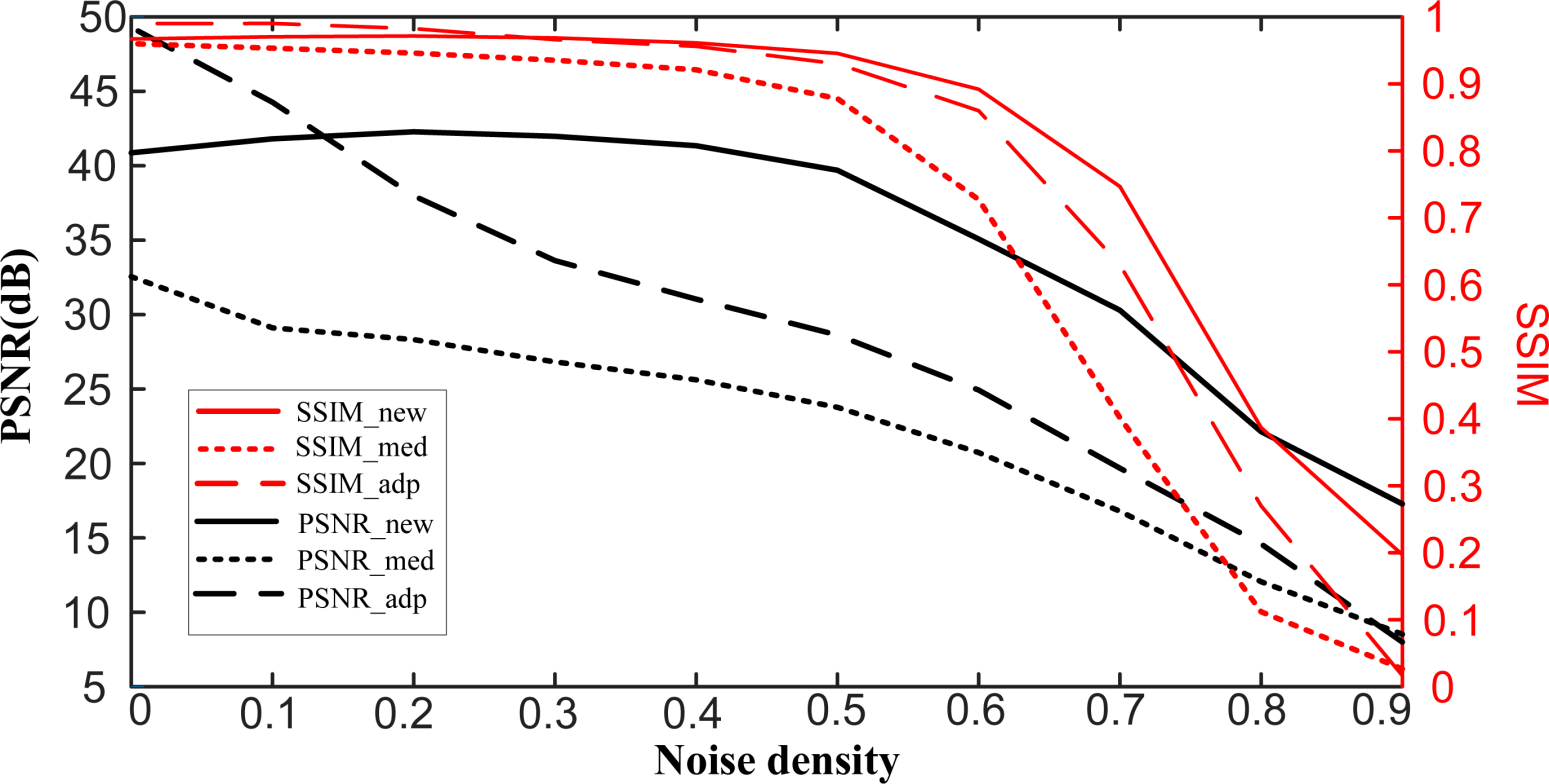
Denoised results comparison of the proposed method, the median filter and the adaptive median filter under different noise density.

AFM images with added noise of different densities are processed by the self-comparison-BCS denoising to evaluate its robustness (Figure 6). When the noise density is low, both the proposed method and the adaptive method exhibit excellent denoising performance. Although the denoising performance of the adaptive median filter is better than that of the proposed method when the noise density is low, its performance drops sharply with increasing noise density. In addition, impulse noise filtering methods using machine learning [10], support vector machines [38], or neural networks [12] encounter the same problem as the adaptive median filter. When the noise density is lower than 0.5, the values of PSNR and SSIM acquired by the proposed method remain almost constant, with PSNR values of more than 40 dB and SSIM values of more than 0.9. That is to say, a high-quality denoised image with negligible distortion and high visual quality can always be obtained, regardless of the noise density. The proposed method shows a more stable performance than the adaptive median filter, and its denoising performance is better than that of the adaptive median filter when the noise density increases.

As the noise density continues to increase, the denoising performance of the proposed method will become worse. This is consistent with the theory that CS requires the sampling rate to be not less than the lower limit to guarantee reconstruction images without distortion [14], which can be described by

[7]
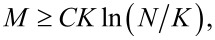


where *M* is the number of noise-free pixels, *K* is the sparsity of the signal, 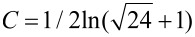, and *N* is the total number of pixels. When the noise density is low, only a small number of pixels will be removed, which guarantees *M* ≥ *CK* ln(*N*/*K*). As the number of noisy pixels continues to increase, more and more pixels are removed from the image. When the number of remaining pixels *M* is less than *CK* ln(*N*/*K*), a decline of the quality of the image is inevitable. The robustness of the proposed method allows it to be used to remove impulse noise with different densities from AFM images. In addition, the erroneous removal of noise-free pixels by the proposed method can be regarded as reducing the sampling rate. A high-quality image can be obtained as long as the number of pixels is greater than *CK* ln(*N*/*K*). Therefore, erroneous removal of partial noise-free pixels will hardly affect the denoising performance. The proposed method will have a more stable performance in the removal of impulse noise than replacing the noisy pixel with the median value.

**Figure 7:**
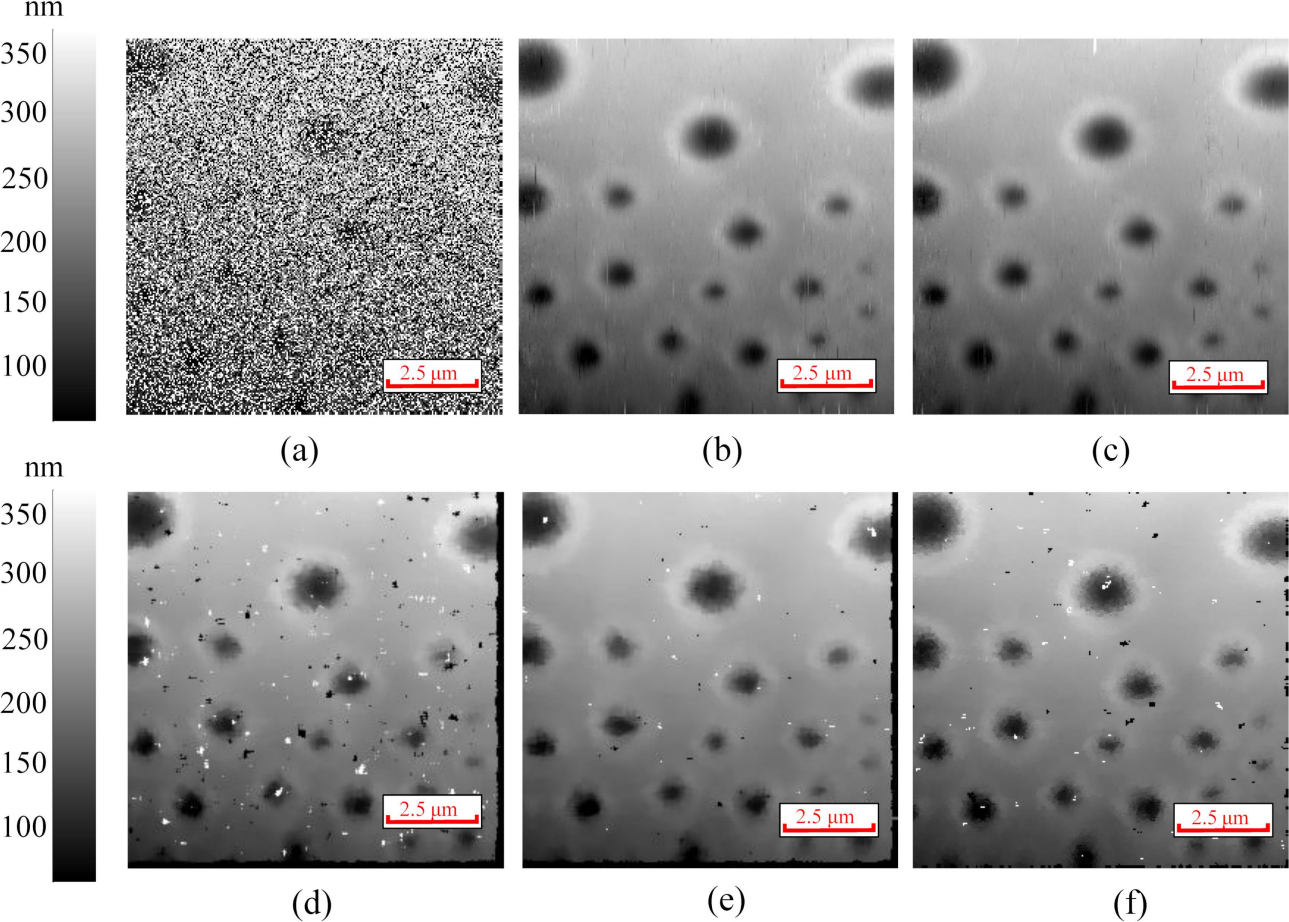
(a) AFM image with added noise of high density. (b) Denoised image obtained by the interval-BCS denoising. (c) Denoised image obtained by the self-comparison-BCS denoising. (d) Denoised image obtained by the median filter with a filter window of 6 × 6 pixels. (e) Denoised image obtained by the median filter with a filter window of 7 × 7 pixels. (f) Denoised image obtained by the adaptive median filter with a maximum window size of 7 × 7 pixels and a minimum window size of 3 × 3 pixels.

**Figure 8:**
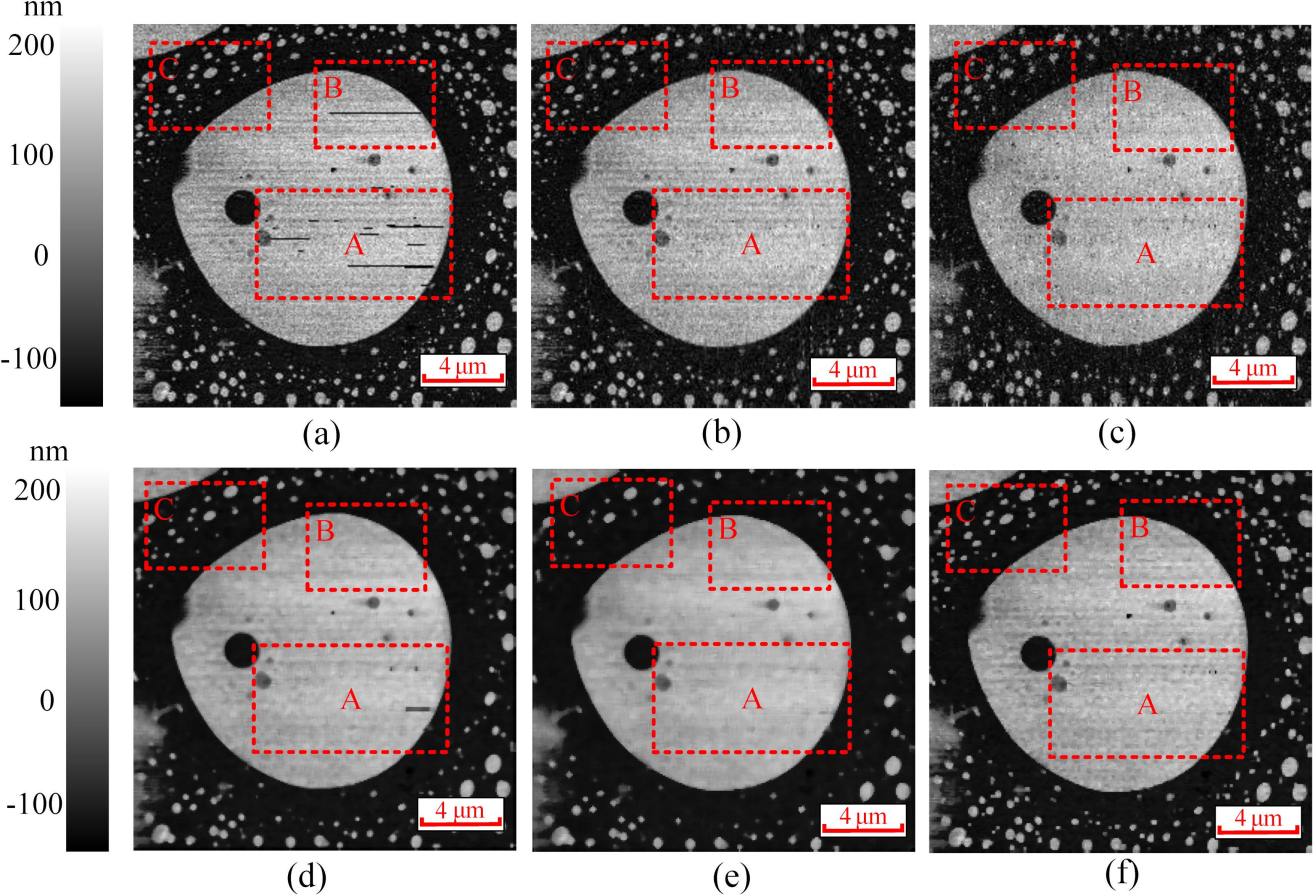
(a) AFM image with added noise. (b) Denoised image obtained by the interval-BCS denoising. (c) Denoised image obtained by the self-comparison-BCS denoising. (d) Denoised image obtained by the median filter with a filter window of 4 × 4 pixels. (e) Denoised image obtained by the median filter with a filter window of 5 × 5 pixels. (f) Denoised image obtained by the adaptive median filter with a maximum window size of 7 × 7 pixels and a minimum window size of 3 × 3 pixels.

To further evaluate the performance of the proposed method, an AFM image with a high density of added impulse noise (Figure 7) and an image of a polymer-blend sample (stiff polystyrene (PS) and soft polybutadiene (PB) polymer, Nanosurf) with noise acquired with a Park Systems XE-100 AFM (Figure 8) are processed by the proposed method. It can be seen from Figure 7 that the performance of the proposed method is better than that of the median filter and the adaptive median filter. In addition, the noise can be seen clearly in the areas (A) and (B) of Figure 8a. The interval-BCS denoising (Figure 8b), the self-comparison-BCS denoising (Figure 8c), the median filter (Figure 8d,e) and the adaptive median filter (Figure 8f) are used to remove the noise. Comparing Figure 8a with Figure 8d, the noise in the area (A) is not removed completely. Although the noise in the areas (A) and (B) is removed completely after median filtering, the small PB islands in area (C) disappears along with the noise, which means a loss of details. The images obtained through median filter look smoother, but all details of the images are sacrified. There are more details reserved in the Figure 8f compared with the images obtained by the median filter. However, the proposed method reserves more details (small dots in area (C)) of the image.

## Conclusion

A novel method to remove impulse noise from AFM images based on the Bayesian compressed sensing is proposed, which transforms the image denoising problem to a compressed sensing imaging problem of the AFM. First, two different ways, interval approach and self-comparison approach, are proposed to identify the noisy pixels. An undersampled AFM image can be obtained by removing the identified noisy pixels. Second, a series of measurement matrices is constructed by recording the position of the remaining pixels. Third, the BCS reconstruction algorithm is used to recover the image, and each row of the AFM image is reconstructed separately. The denoising experiments demonstrate that the proposed method can remove impulse noise from AFM images while preserving the details of the images. Upper and lower limits are key parameters for the interval-BCS denoising. The self-comparison-BCS denoising identifies noisy pixels by comparing a target pixel with its neighborhood. However, the performance of the self-comparison-BCS denoising is worse than that of the interval-BCS denoising when the image contains a lot of flat areas and periodic structures. The proposed method is robust because its performance remains stable in a certain noise density range, and the erroneous removal of few noise-free pixels hardly affects its performance. Therefore, the proposed method can be used to remove the impulse noise from AFM images with different noise densities without worrying about the degradation of the final image quality. The proposed method is an effective, competitive and robust method to remove impulse noise from AFM images.
